# Do advanced glycation end products contribute to food allergy?

**DOI:** 10.3389/falgy.2023.1148181

**Published:** 2023-04-04

**Authors:** P. K. Smith, C. Venter, L. O’Mahony, R. Berni Canani, O. J. L. Lesslar

**Affiliations:** ^1^ Clinical Medicine and Menzies School of Research, Griffith University, Gold Coast, QLD, Australia; ^2^ Children’s Hospital Colorado, University of Colorado, Aurora, CO, United States; ^3^ Department of Medicine, School of Microbiology, APC Microbiome Ireland, University College Cork, Cork, Ireland; ^4^ Department of Translational Medical Science and ImmunoNutritionLab at CEINGE-Advanced Biotechnologies, University of Naples “Federico II”, Naples, Italy; ^5^ Cingulum Health, Sydney, NSW, Australia

**Keywords:** food allergy, alarmin, advanced glycation end products, receptor for advanced glycation end products (RAGE), high molecular group box 1, carboxymethyllysine (CML), methylglyoxal (MG)

## Abstract

Sugars can bind non-enzymatically to proteins, nucleic acids or lipids and form compounds called Advanced Glycation End Products (AGEs). Although AGEs can form *in vivo*, factors in the Western diet such as high amounts of added sugars, processing methods such as dehydration of proteins, high temperature sterilisation to extend shelf life, and cooking methods such as frying and microwaving (and reheating), can lead to inordinate levels of dietary AGEs. Dietary AGEs (dAGEs) have the capacity to bind to the Receptor for Advanced Glycation End Products (RAGE) which is part of the endogenous threat detection network. There are persuasive epidemiological and biochemical arguments that correlate the rise in food allergy in several Western countries with increases in dAGEs. The increased consumption of dAGEs is enmeshed in current theories of the aetiology of food allergy which will be discussed.

## Introduction

A Western diet has been linked to an increasing rate of food allergy (1). This is also intertwined with dietary inadequacy and the trend of increasing sugar intake, particularly fructose, in the last 50 years in the human diet (2, 3). Sugars can bind to proteins, nucleic acids or lipids, and form compounds called Advanced Glycation End Products (AGEs). This process does not involve enzymes (4, 5). Although AGEs can form *in vivo*, factors in the Western diet such as high amounts of added sugars, processing methods such as dehydration of proteins, high temperature sterilisation to extend shelf life, and cooking methods such as frying and microwaving (and reheating), can lead to inordinate levels of dietary AGEs. Dietary AGEs (dAGEs) have the capacity to bind to the Receptor for Advanced Glycation End Products (RAGE). RAGE is a part of the endogenous threat detection network as it can be activated by amyloid and Danger Associated Molecular Patterns (DAMPs) including High Molecular Group Box 1 (HMGB1) and S100 proteins (6). RAGE agonism induces several intracellular proinflammatory processes (7). There is a persuasive argument that correlates the rise in food allergy in several Western countries, with increases in dAGEs. This is supported by epidemiological data and by advances in our understanding of the immunological processes that the AGE-RAGE axis influences (8–12). The increased consumption of dAGEs is enmeshed in current theories of the aetiology of food allergy, including the hygiene hypothesis (13), the role of the gut microbiome in food allergy (14), epidermal barrier dysfunction (15), lack of dietary diversity (16), dietary fiber (17), low vitamin D (18), and delayed introduction of high-risk foods in the first year of life (19, 20). This review brings together data, concepts, and findings from *in vitro*, animal models, atopic and inflammatory diseases other than food allergy. HMGB1 is the archetypal alarmin and while AGEs bind to the same receptor, there remain knowledge gaps as to whether dAGEs have identical actions as HMGB1 in its mechanisms in food allergy.

## Advanced glycation end products

Glycation refers to the bonding of saccharides (fructose, glucose, galactose or ribose) to a protein, nucleic acid or lipid molecule without enzymatic regulation (4, 5). The most familiar process is the Maillard reaction, where foods are browned with heating, and resultant downstream compounds include Methylglyoxal (MG) and Advanced Glycation End Products (AGEs) such as carboxylmethyllysine (CML) (5). The Western diet and increasing use of processing methods utilised globally in ultra-Processed Foods (UPF) production, promote the formation of dietary AGEs (dAGEs). The Western diet and UPFs are typically high in sugar content, use dehydrated ingredients, employ very high cooking temperatures (microwaving or frying), and have long shelf lives (21–23). AGEs can also form endogenously (eAGEs) and signal *via* the AGE receptor (RAGE) to initiate a danger pattern (24). Similarly, certain microbiota can produce AGEs (mAGEs) (25), which can contribute to inflammatory responses, and should be thought of as part of the total AGE pool.

## AGE receptors

Unless otherwise specified, this paper will refer to full-length RAGE, which comprises intracellular, transmembrane and extracellular domains. In addition to this archetypal RAGE there are 19 reportedly splice variants (26). A splice variant, lacking the intracellular and membrane domains generate a soluble version of the receptor (sRAGE) that provides a natural decoy for potential RAGE agonists. The importance of RAGE agonism in shaping allergic responses is underpinned by serum levels of sRAGE being protective for asthma (27) and higher levels of sRAGE have been associated with lower levels of serum IgE in asthma (28).

**Figure 1:**
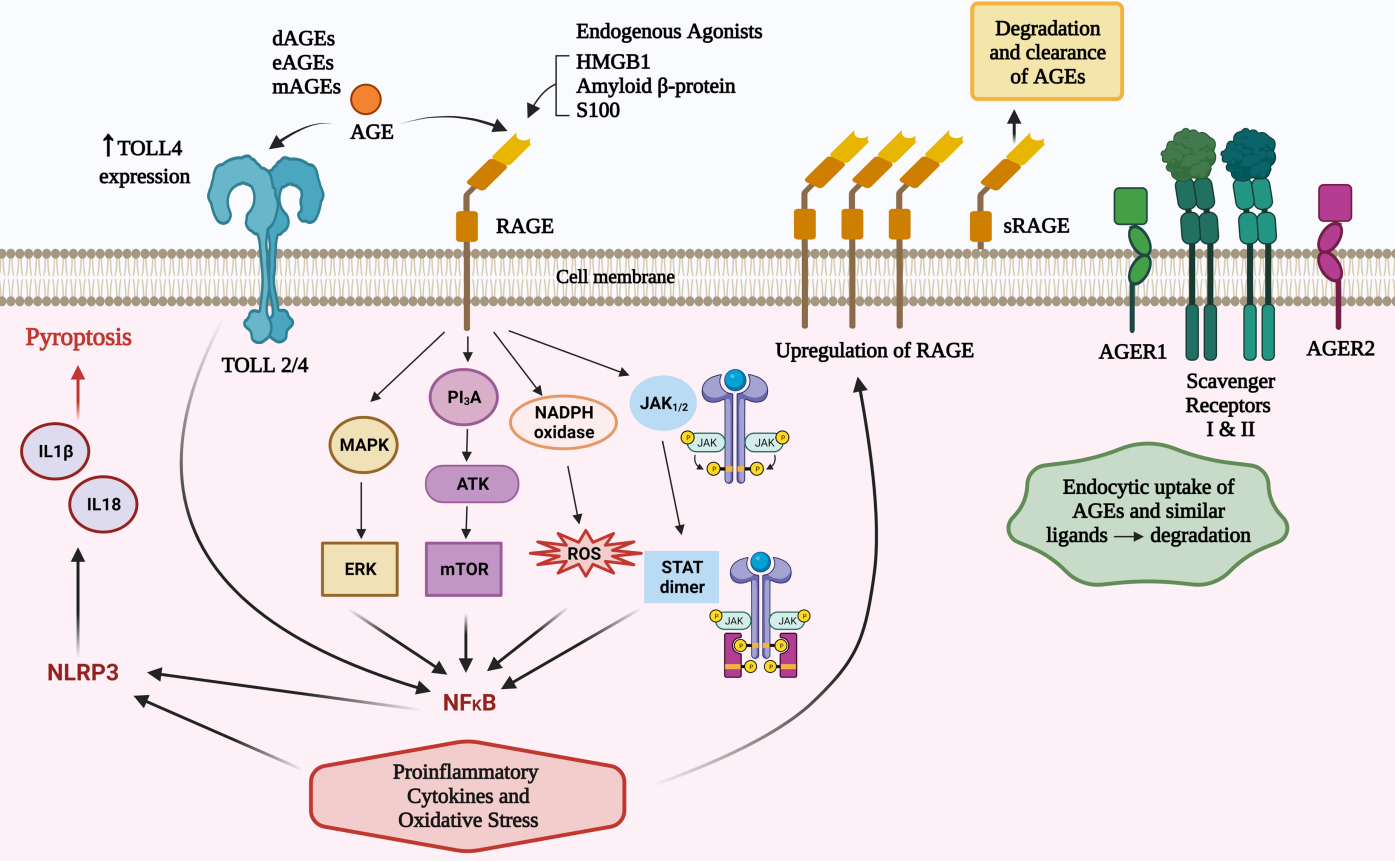
Total body pool AGEs comprise those from diet (dAGEs), endogenous (eAGEs) and microbial (mAGEs). These bind to AGE receptors (RAGE) to activate several pathways that contribute to inflammatory responses. AGEs can increase TLR 4 receptor expression and there are converging pathways with TLR activation that may amplify RAGE agonism. There are other AGE ligands that have a role in clearance of these potentially inflammatory compounds.

Other RAGE worth noting are oligosaccharyltransferase 48 (AGER1), 80 K-H phosphoprotein (AGER2), galectin-3 (AGER3), and type I and II scavenger receptors (29). AGER1 and the scavenger receptors have a capacity to not only bind to AGEs and similar ligands, but also transport them intracellularly and degrade them (29) (summarised in Figure 1). It is plausible that defects of this degradation process may result in higher levels of RAGE agonists and increased atopic influences. This concept has not been explored in research in any inflammatory disease to date, however it is worthy of consideration due to the protective effect on atopy by higher levels of sRAGEs.

RAGE agonism can activate nuclear factor kappa-light-chain-enhancer of activated B cells (NF-κB) *via* four main mechanisms (PI3K/AKT, MAPK/ERK, JAK2–STAT1, and NADPH oxidase) (5, 30). RAGE agonism induces intracellular proinflammatory processes leading to multiple types of cellular activation, cytokine responses, pyroptosis and inflammaging (7). The inflammatory cytokines induced can influence innate and subsequent adaptive immune responses (5, 7, 30) including an amplification loop *via* increased expression of the RAGE receptor (7); activation of NADPH oxidase to cause reactive oxidative and nitrogen intermediates; mitochondrial and endoplasmic reticulum stress (5, 7, 30), and *via* NLRP3 inflammasomes, production of IL18 and IL1*β* which can cause cellular death *via* pyroptosis (31). TLR2/4 can be increased by AGEs and there are shared intracellular signalling pathways that can augment AGE and TLR2/4 signalling (32) (Figure 1).

## Potential dAGE-driven cellular mechanisms of allergy

RAGE is part of the endogenous threat detection network as it can be activated by amyloid and Danger Associated Molecular Patterns (DAMPs) including High Molecular Group Box 1 (HMGB1) and S100 proteins (6). AGE ligation and the inflammatory responses implicating dAGEs in food allergy, include their ability to injure the gut epithelium (33, 34) which promotes inflammation and altered antigen presentation—conditions unfavourable to maintain dietary tolerance.

Dendritic cells (DCs) have at least 6 RAGE receptors (RAGE, AGER3, AGER1 and type I and II scavenger receptors, and CD36 (35). The scavenger receptors are reported to have a role in processing of antigens by way of endocytosis (36). DCs produce HMGB1 which is critical for dendritic cell maturation, activation, antigen processing and presentation to T cells (35, 37–40). *In vitro* studies show that AGE-stimulated human dendritic cells lead to greater Th2 responses and increased expression of RAGE (41). HMGB1 can directly act on naive CD4+ T cells to induce differentiation of Th2, Th17 cells *in vitro* through activating the TLR2, TLR4, and RAGE-NF-κB signal pathways (42). It has been proposed that RAGE may contribute in part to polarisation of CD4 + lymphocytes and the balance of Type 1 and 2 lymphocytes (43–46).

High serum levels of HMGB1, which dAGEs can mimic, have been linked to higher serum IgE levels (47). IgE can be evoked in response to roasted peanuts and their specifically modified Advanced Glycation End Products to RAGE, as opposed to raw peanuts (48). Higher levels of RAGE expression on T and B lymphocytes are strongly associated with activity and inflammatory responses of these cells (49).

**Figure 2:**
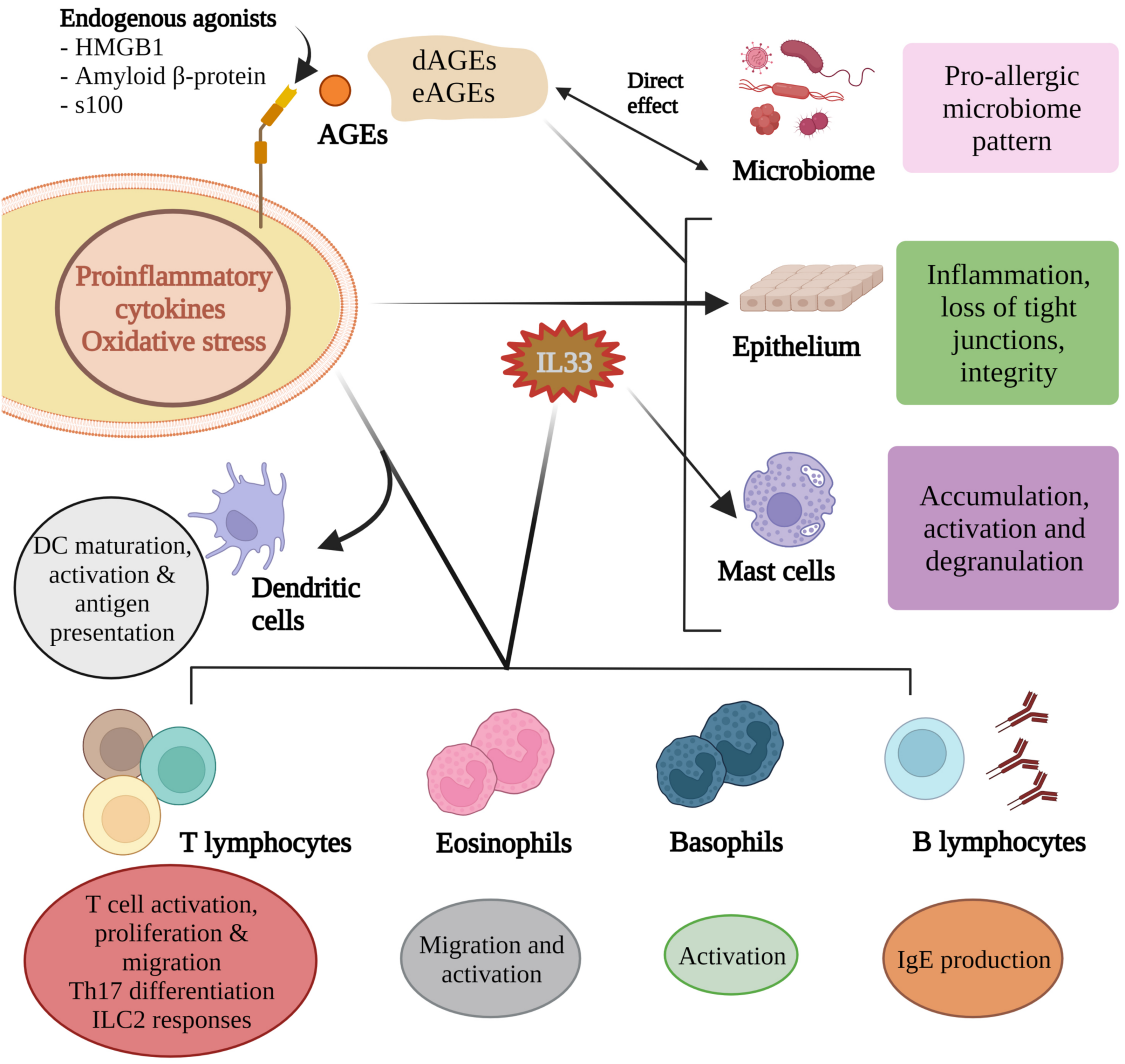
Advanced glycation end products influence microbial composition and activity and these in turn partly contribute to the AGE pool. AGEs can directly influence epithelium integrity as can the inflammatory mediators induced by RAGE agonism. Of the many pro-allergic cytokines produced, IL33 helps shape the allergen responses of B lymphocytes, mast cells, eosinophils and basophils. RAGE agonism is critical for dendritic cell maturation, activation and antigen presentation, T cell responses to allergens, and Th17 differentiation.

AGEs activate mast cells to release proinflammatory mediators (50). HMGB1 has been demonstrated to increase mast cell accumulation (51). Albeit in an airway model, AGE activation in response to an allergen promotes IL33 production and subsequent activation of basophils, mast cells and eosinophils (42, 52), and accumulation of group 2 innate lymphoid cells (51) (Figure 2). High amounts of dAGEs may exceed the body's capacity to degrade AGEs (on top of regular physiologic eAGEs load) which is usually maintained by sRAGE, AGER1 and scavenger receptors I and II (7).

## The Western diet and the rise of food allergy

Diets in the modern era have changed dramatically in the last 50 years and the Western diet pattern of eating has been linked to an increasing rate of food allergy (1). This is also intertwined with dietary nutritional inadequacies., The trend of increasing sugar consumption, particularly fructose, an ever-increasing reliance on UPFs in the last 50 years (2, 3), and the decrease in the diversity of the gut microbiome (53). Bach's et al. (54) notably associated reduced severe microbial infections with an increase in both Th1 and Th2 disease, implying a role for microbial challenges to our resident microbiome to improve immunity. In a 2022 update, Larsen et al. (55), provided more support for these findings, reporting that the incidence of prototypical autoimmune diseases anti-correlated with the incidence of common infectious diseases. Furthermore, Larsen and colleagues showed increases additional increases in metabolic and autoimmune disease coincided with increases usage of antibiotics and emphasised that the status of the gut microbiota is persistently deteriorating (55).)**. Several studies have discussed reduced microbial infections and time trends in T1 diseases as well as T2 conditions of eczema, food allergy, and anaphylaxis discharge data (9, 10, 56–58). Smith et al. (9, 10) point to a rise in childhood allergies and correlated types of foods consumed by young children in the US (59) and patterns of fast-food consumption in Australia (9) with the rise in food allergies.

A global review found that intake of added sugars was higher in school-aged children and adolescents (up to 19% of total energy) compared to younger children or adults (60). The same authors revealed a 3-fold increase in sugar consumption within a 15-year period, and strikingly 2–3 year old Australian children were consuming 90 grams of sugar per day in 2011. Each of the countries in the review have strong epidemiological data indicating an increasing rate of food allergy and anaphylaxis (61–65). Causation cannot be established, but this does raise the question of the role of sugar in food allergies, likely in part through the formation of AGEs. High free sugar intake in pregnancy has been linked in a meta-analysis to increased offspring risk of asthma, allergic rhinitis and food allergy (66). High-fructose corn syrup (HFCS) comprises 55% fructose and 45% glucose. Fructose can cause an elevation in uric acid (67), which is a non-RAGE inducing alarmin. Soft drink comprises over 40% sweeteners (68). The Western diet contains more than 2000% HFCS compared to more traditional diets (69). Dietary consumption of free fructose is associated with an increased risk of allergic sensitisation and symptoms (70). Adolescents consuming beverages high in fructose 5 times a week or more have a five-fold risk of having allergic symptoms compared to those who reported infrequent consumption of these beverages (70). Uric acid augments Th2 allergic inflammation (71, 72) and high uric acid levels have been linked to peanut allergy *via* dendritic cell activation (73). Fructose, compared to glucose, forms several-fold higher level of AGEs (74). Fructose may also be formed intracellularly from glucose *via* the polyol pathway involving aldose reductase and sorbitol dehydrogenase (75). The latter enzymatic pathway is also a method for formation of AGEs from xylose which is artificially extracted from glucose (76). Xylose has an even-greater capacity than fructose to form AGEs (77).

Modern common table salt—sodium chloride—is found in abundance in processed foods (78). This is in stark contrast to traditional diets where salts were a full spectrum of electrolytes and rich in minerals (79). Excess sodium chloride activates the aldose reductase pathway leading to greater formation of intracellular fructose and AGEs (80). Intracellular AGEs are associated with glycation of intracellular proteins, cellular dysfunction, cell cycle arrest (81), disturbed DNA repair, and inhibition of the glyoxalase system (82). The glyoxalase system comprises enzymatic mechanisms for degrading MG and other glycation intermediary compounds (83). DNA damage by MG has been reported to be increased with vitamin B9 deficiency (84). Vitamin B9 (folate) is found in fresh fruits and vegetables and insufficiency of folate in the population, even with government-driven dietary folic acid (synthetic vitamin B9) fortification, is common in Western countries (85, 86).

The Western diet is commonly associated with UPFs, foods that are dehydrated and having long shelf lives, and food preparation methods like microwaving or frying, all of which increase the formation of AGEs (23, 87, 88). Uribarri et al. showed that microwaving increased dAGEs in milk and this increased exponentially with time; from 1 AGE kU/250 ml serving without heating to 5, 19 and 80 at 1, 2 and 3 min of microwaving respectively (87). This indicates higher dAGE consumption with microwave heating and re-heating of foods.

Heating and the formation of AGEs within food does not always results increased allergenicity. Heating and the formation of AGEs in food does not always result in increased allergenicity. High levels of glycation of bovine lactoglobulin, a common cow's milk protein allergen, results in alteration of the molecular allergen binding sites and less IgE binding (89).

The consumption of increasing amounts of dietary sugars and fast food is to be factored with studies showing that there is ingestion of 7–8 kg per person per year of synthetic chemical additives including preservatives, acidity regulators, colorants and emulsifiers (90). These additives have been linked to allergic outcomes, most likely *via* their alteration of microbiome composition and function (91, 92). The Western diet has less omega 3 fatty acids (93) and dietary fiber (94), both of which have been suggested to further contribute to risk of allergic disease (95, 96). The correlation between dAGEs and allergy has multiple intersection points in the Western diet.

## Epidemiological associations

As already outlined, countries such as Australia and the United States, increasing uptake of highly sugared foods, UPFs, and fast food which is mostly fried, correlates with an increase in severe food allergy and anaphylaxis (9, 10). We are seeing that countries adopting these dietary trends are also observing an increase in chronic poor health conditions, including allergies and obesity (97). Conversely, a traditional Mediterranean diet, which is not just associated with less sugars and dAGEs, but also with more omega 3 fatty acids and plant fibers. Omega 3 fatty acids and dietary fiber leads to enhanced metabolism of AGEs, greater antioxidant capacity, a preferential gut microbiota, and more resilient mitochondria, which have all been linked with a reduced risk of chronic disease and allergies (83, 87, 98–100). A Mediterranean diet includes the liberal use of herbs such as garlic and rosemary, and features more slow cooking at lower temperatures, all of which reduce the formation of AGEs (87). Reports link the Mediterranean diet with a reduction in wheeze, rhinitis and IgE-mediated sensitisation (101–103).

Urban dwelling populations consume soft drinks and confectionery at levels up to twice that of their rural counterparts (2, 104), a trend demonstrated in Australia where children living in urban areas are also more likely to eat fast food, and more likely to be obese (105). Urban living compared to rural living, in the USA, has been linked to a doubled risk of peanut and shellfish allergies (106). We assert that in addition to environmental factors and gut microbiome diversity contributing to reduced risk of allergy in rural settings (107–109), more fresh, whole foods and less dAGE burden are also important factors. “Food deserts” describe urban areas where fresh food is hard to obtain, more fast food is consumed, and there are greater rates of obesity. A review of ZIP codes in the US identified that a child living in a food desert vs. a non-food desert had a risk ratio of 1.56 for food allergy (110).

Hyperglycemia is a risk factor for the formation of dAGEs. Elevated maternal blood glucose in pregnancy has been associated with an increase rate in IgE sensitization OR 1.6 (driven by food sensitization) and atopic dermatitis OR 1.7 in US women (111). This finding was in term infants rather than preterm infants. 20% of pregnant women in the USA are obese (112) and obesity is strongly related to decreased diversity of the gut microbiome and metabolic health (113). Western women are having children later in life and the glyoxalase system reduces with chronological age so this may also be a factor with this pregnancy association (114). A recent 2021 study from the US showed significant associations between maternal AGEs intake during pregnancy and offspring allergy outcomes or cord blood cytokines and chemokines (115) although a 2010 study showed no association between maternal pregnancy dietary patterns and recurrent wheeze in their offspring (101).

Despite many researchers' efforts, the AGEs content of many foods is unknown and calculation of the AGEs scores of composite foods is difficult to standardize. Future studies may benefit from using an AGEs food frequency questionnaire, validated against reliable biomarkers such as serum levels of AGEs. This information will provide us with validated measures of dietary intake (115).

## AGEs and existing theories of food allergy

### The microbiome

Several hypotheses have been proposed that link allergic disease risk with the microbiome, or more specifically a depletion thereof, such as “missing microbes”, “microbiome depletion”, “microbiome diversity”, “microflora”, “overarching microbiome”, and “old friends” hypotheses (116). The Western diet, UPFs, low plant fiber intake, and urban living are associated with less microbial diversity and more inflammation (117, 118), so the contribution of each of the factors still needs to be determined in the development of food allergy. dAGES disrupt and alter the composition of the gut microbiome and their products, contributing to inflammation (25). Only 10%–30% of dAGEs are absorbed, meaning that over 70% interact with the colonic epithelium and microbiome until being passed out in the stool (119). Studies on CML suggest 20%–50% of this AGE is excreted in faeces indicating a metabolic interaction with microbiome (120). Many dAGEs can be degraded by gut microbiota to provide a source of nitrogen for growth (121).

Certain bacteria can generate mAGEs, for example *Escherichia coli* secrete mAGEs by the energy-dependent efflux pump system and uses this as a local toxin to reduce growth of neighboring competitive bacteria, thus having local inflammatory effects, as well as influencing the microbiome composition (25). *Bifidobacteria* and *Lactobacilli* are amongst a group of bacteria that produce the MG degrading enzyme glyoxalase-1, which can protect from bacteria-produced AGEs and is also capable of degrading dAGES (122–124). Evidence for dAGEs impacting microbial composition include studies that show markedly reduced Bacteroidetes/Firmicutes ratio and increased inflammatory markers including IL-1β, IL-17 and Plasminogen activator inhibitor-1, and increased incretins such as gastric inhibitory polypeptide (GIP) and glucagon-like peptide-1 (GLP-1) exacerbating insulin resistance (125–127). These may increase serum blood glucose levels and further amplify formation of eAGEs, contributing to obesity. dAGES have been associated with greater protein fermentation, associated by increased levels of the putrefactive toxic metabolites, ammonia and branched chain fatty acids at the expense of reportedly beneficial metabolites like short chain fatty acids (SCFAs) which are produced when fiber is fermented in the colon (128). Butyrate, a SCFA, has been linked with both protection against food allergy as well as having benefit in oral immunotherapy for food allergy (129, 130). Microbiota species including *Lactobacilli*, *Bifidobacterium* and *Prevotella* have been linked to protection from development of food allergy and these microbiota demonstrably decrease with dAGEs (131–134). We direct readers to a recent review by Phuong-Nguyen et al., on the effect of AGEs on the gastrointestinal tract (135).

### Barrier function

It has been hypothesized that epidermal sensitisation *via* an inflamed and/or disrupted skin epithelium can result in sensitisation and allergy to foods (136), and this is correlated with evidence that increased epithelial permeability at birth has been associated with risk of food allergy at 2 years of age (15). Several studies have demonstrated the bidirectional link between gut dysbiosis and skin homeostasis imbalances (137, 138). Studies indicate that there is increased pathological gastrointestinal permeability in children with atopic eczema (139).

In an animal model, a high dAGE intake led to loss of epithelial tight junctions (claudin-1 and 5, occludin) in the jejunum, ileum and colon, and subsequent increased gut permeability (140). This has been confirmed in other animal studies looking at dAGEs and HMGB1, however their focus had been on colonic epithelial integrity (33, 141). AGEs can also increase epithelial-produced cytokines which in turn can create an inflammatory milieu for antigen processing. This inflammation may contribute to loss of epithelial barrier function. *In vitro* studies looking at the effect of glycated dairy-derived caseinates demonstrate increased permeability and loss of tight barrier function (34, 142). There is evidence of epithelial injury by AGEs influencing the microbiome, this is partly by way of a reduction in butyrate-producing bacteria which normally bolsters colonic epithelial barrier integrity (143).

### Vitamin D

Low vitamin D levels have been linked to increased risk of food allergy and anaphylaxis, evidenced by epidemiological data indicating that living further away from the equator can increase the risk of peanut allergy by up to 6-fold (144–147). Birth in winter and spring (associated with lower levels of vitamin D in mother and offspring) is associated with increased risk of food allergy (148, 149). Vitamin D deficiency can result in higher expression of the RAGE, lower levels of sRAGE and higher serum levels of glyoxalase I enzyme (150). It is notable that *Lactobacillus reuteri,* a species associated with reduced Th2 responses (151), is capable of increasing serum vitamin D (152). Vitamin D is also involved in microbial TLR signalling (153). There is convergence of RAGE and TLR 2/4 pathways to induce inflammation, and HMGB1 is capable of agonising these specific TLRs (32).

The composition of the gut microbiome can be altered by vitamin D status/ sun exposure. Human studies have reported significant associations between vitamin D and microbiome composition. It has been well-demonstrated that vitamin D is necessary for gastrointestinal barrier integrity by effects on tight junction proteins, and reducing epithelial apoptosis (154–156). As mentioned previously, a loss of microbiota diversity and an increase in barrier dysfunction both contribute to allergy risk (154).

### Early complementary feeding

Studies introducing peanut and egg in infancy have been shown to reduce risk of allergy to these foods (19, 32). In the following two studies: Learning Early about Peanut Allergy (157), and Iannotti's early introduction to egg study (158), sub-analyses indicated that the intervention groups developed less allergy, and had also consumed less sweetened and processed foods, querying the role of decreasing dAGEs in the reduction of food allergy. Intervention studies in food allergy should consider analyses of dAGEs.

## Reducing AGEs

The potential adverse health effects of AGEs go beyond allergic risk, extending to cardiac, renal and brain disease as well as “inflammaging” (159). Interventions to reduce dAGEs can be relatively easily achieved with lifestyle interventions (159, 160). These include avoiding fried and microwaved foods, as well as sugars and sweetened beverages (including commercial fruit juices) (87, 161), improvements in circulating AGEs, inflammatory markers, and insulin resistance may be seen within 4 weeks (162). Cooking with herbs and spices, such as rosemary, garlic, star anise, ginger, cloves, cinnamon and allspice reduces the formation of AGEs (163–165). We direct the reader to Uribarri's practical guide to reducing dietary AGEs (87) It is worth noting some foods regarded as having health benefits may be relatively high in dAGEs. Caloric restriction has been shown to reduce circulating AGEs (166, 167), however the mechanisms by which this occurred has not been elucidated. The mechanisms of this have not been elucidated however, lower endogenous AGEs will be formed with lower serum glucose levels and less oxidative stress, there will be a reduction in formation of AGEs. Enteric microbial activity will be reduced in fasting states so that mAGEs formation should be less. Conversely, a single high fat meal has been associated with increased cellular RAGE and reduced sRAGE (168). Kim et al. (169) has suggested that regular physical activity can attenuate the effect of AGEs by its antioxidant mechanisms, reduction of ROS, and reduction of newly formed AGEs *via* better glycaemic control. A Japanese study reported lifestyle factors including stress, lack of exercise and inadequate sleep were associated with higher measurements of AGEs (170).

Histamine is derived from the amino acid histidine, mediated by the enzyme l-histidine decarboxylase. Histamine exerts immunoregulatory effects and along with its receptors, are involved in food antigen tolerance and mediate the symptoms of intolerance, sensitivity, and allergy (171). L-histidine decarboxylase uses pyridoxal phosphate (vitamin B6) as a cofactor (172). An RCT of adult asthmatic patients showed vitamin B6 levels were associated with decreased rates of asthma symptoms and exacerbations (173). In a double-blind study of 76 asthmatic children, vitamin B6 supplementation was associated with improvement in asthma symptoms and consequent reduction in asthma medication-use (174). Vitamin B6 has also been shown to inhibit the AGE formation pathway (175).

Vitamin C appears to prevent the secretion of histamine by white blood cells and increase its metabolism (176). Histamine levels were found to increase exponentially as ascorbic acid levels in the plasma decreased (177). Vollbracht et al. (178) demonstrated that high doses of intravenous vitamin C had positive clinical benefits for patients with both acute and chronic allergic rhinitis. Vitamin C has been found to inhibit glycation of serum bovine albumin by 52% and inhibits biochemical reactions important in decreasing AGEs, including the production of oxygen-derived free radicals, the accumulation of sorbitol within cells, and tissue-damaging glycosylation (179).

Berberine has been shown to reduce Th2 responses in an allergic airways disease model (180) and has also been used to help induce tolerance of allergic foods (181). In a rat model, cells treated with berberine showed reduced levels of AGEs, accompanied by decreased RAGE levels soon afterwards (182). In a recent 2021 study, berberine was described as a potent AGEs inhibitor, significantly suppressing AGE formation in retina endothelial cells (183).

## Conclusion

Factors in the Western diet such as high amounts of sugars, processing methods such as dehydration of proteins, high temperature sterilisation to extend shelf life, and cooking methods such as frying and microwaving (and reheating), can lead to very high levels of dAGEs. There are suggestive epidemiological and compelling immunological mechanisms which associate AGEs with increased risk of allergy. dAGES and RAGE activation disrupt and alter gut epithelial barriers and the composition of the gut microbiome and their products. These lead to inflammatory responses that can drive food allergy, as there is further injury to the gut epithelium, further inflammation and altered antigen presentation. RAGE may contribute in part to polarisation of CD4 + lymphocytes and the balance of Type 1 and 2 lymphocytes. In sum, these are all conditions unfavourable for dietary tolerance. Multiple dietary modifications and lifestyle interventions have the potential to reduce the formation of AGEs, mitigate their oxidative effects, and reduce expression of the RAGE receptor.
